# High prevalence and incidence of rectal *Chlamydia* infection among men who have sex with men in Japan

**DOI:** 10.1371/journal.pone.0220072

**Published:** 2019-12-10

**Authors:** Daisuke Mizushima, Misao Takano, Haruka Uemura, Yasuaki Yanagawa, Takahiro Aoki, Koji Watanabe, Hiroyuki Gatanaga, Yoshimi Kikuchi, Shinichi Oka

**Affiliations:** AIDS Clinical Center, National Center for Global Health and Medicine, Tokyo, Japan; University of Texas Health Science Center at San Antonio, UNITED STATES

## Abstract

**Background:**

Rectal *Chlamydia trachomatis* (CT) and *Neisseria gonorrhoeae* (NG) infections have been neglected and epidemiological data are unavailable in Japan. Thus, we evaluated the prevalence and incidence of rectal CT/NG in a cohort of HIV-negative men who have sex with men (MSM), which was established at the National Center for Global Health and Medicine (NCGM), in Tokyo, Japan, in January 2017.

**Methods:**

HIV-negative MSM aged ≥16 years old were included. The prevalence of rectal CT/NG among HIV-negative MSM was compared with that among an existing HIV-positive MSM cohort at NCGM. The HIV-negative MSM cohort was examined for rectal and pharyngeal CT/NG every 3 months. Urethral CT/NG was evaluated at the physician’s discretion. The incidences of CT/NG were evaluated in December 2018.

**Results:**

Of 502 MSM initially included in this study, 13 men were diagnosed with HIV infection at enrollment and were subsequently excluded from the analysis. We evaluated 561 HIV-positive MSM for rectal CT/NG. The mean ages of the two cohorts were 33.6 and 46.2 years old, respectively (p<0.001). The prevalences of rectal CT were 16.4% and 15.9% (p = 0.707) and the prevalences of rectal NG were 4.1% and 2.3% (p = 0.101), for the HIV-negative and HIV-positive MSM cohorts, respectively. Of 489 HIV-negative MSM, 328 were followed at least twice, with 261.1 person-years during the study period. The incidences of rectal CT/NG were 17.2 and 3.8/100 person-years and the incidences of pharyngeal CT/NG were 2.0 and 11.0/100 person-years for the two cohorts, respectively. Approximately 37.9% of incident cases were attributed to recurrent infection.

**Conclusions:**

The prevalence and incidence of rectal CT/NG were high among MSM in Tokyo, Japan, suggesting that urgent countermeasures for early diagnosis and treatment are necessary.

## Introduction

Rectal *Chlamydia trachomatis* (CT) and *Neisseria gonorrhoeae* (NG) infections are important since they raise the risk of HIV acquisition[1–4]. Many studies have reported the prevalences of rectal CT and NG among men who have sex with men (MSM) in different settings. The prevalences of these rectal infections vary across studies[5,6]. According to a systematic review that assessed studies mainly from the United States, Australia, the United Kingdom, and the Netherlands, the mean prevalences of rectal CT and NG among MSM were 9% and 6.1%, respectively[7]. Rectal CT infection among women has also become a concern[8,9]. In Japan, unfortunately, rectal sexually transmitted infections (STIs) have been considerably neglected and epidemiological data on rectal CT and NG are not available. Since these rectal bacterial STIs are usually asymptomatic, MSM who are unaware of rectal STIs are likely to transmit CT or NG, and this could be a major challenge in the prevention of such STIs. In terms of HIV prevention, the implementation of pre-exposure prophylaxis (PrEP) is in progress worldwide. Rectal NG and CT are not only risk factors for HIV acquisition but also could be useful markers to identify candidates for PrEP[4]. Conversely, several studies suggested that PrEP implementation might increase the frequency of unprotected anal sexual intercourse and lead to the increased incidence of STIs among people on PrEP[10]. Thus, it is essential to establish a testing system for rectal CT and NG for worldwide implementation of PrEP. In this context, the incidence and prevalences of rectal CT and NG in Japan needs to be determined as well.

To obtain epidemiological data on rectal CT and NG among MSM in Japan, we herein elucidated the prevalences of rectal CT and NG among MSM by establishing a prospective cohort of HIV-negative MSM at the National Center for Global Health and Medicine (NCGM), Tokyo, Japan. In addition, we evaluated incidences of CT and NG in this prospective HIV-negative MSM cohort.

## Methods

### Study design and population

The present study is a single-center prospective cohort study. NCGM is a teaching hospital with 800 beds and the largest referral center for HIV/AIDS in Japan. We established an HIV-negative MSM cohort at the Sexual Health Clinic, NCGM, in January 2017, for PrEP implementation. Inclusion criteria of the HIV-negative cohort were MSM, aged ≥16 years old, who have anal sexual intercourse and gave written informed consent for the study. People who were diagnosed with HIV at enrollment were excluded from the cohort and were referred to a HIV-positive clinic, the AIDS Clinical Center at NCGM, or other medical institutions. In the HIV-negative cohort, all subjects visited the Sexual Health Clinic and underwent HIV screening and serological testing for syphilis and rectal CT and NG every 3 months. After January 2018, pharyngeal CT and NG were added to the routine evaluation for every visit. Between January and March 2018, urinary CT and NG were cross-sectionally evaluated. Furthermore, urinary samples for CT and NG were collected at the physician’s discretion when subjects had urinary tract infection symptoms as required.

HIV-negative and -positive MSM were evaluated for the prevalences of rectal CT and NG. We performed cross-sectional evaluation of rectal CT and NG among HIV-positive MSM at the AIDS Clinical Center between January and March 2017. HIV-negative MSM were enrolled between January 2017 and December 2018, and their rectal CT and NG results at the first visit were used to analyze prevalence. The incidences of rectal CT and NG were also evaluated during the same period.

A nucleic acid amplification test (Bio Medical Laboratories, Inc., Tokyo, Japan) was used to detect CT and NG from clinical specimens of self-collected rectal swabs, mouthwash samples, and urinary samples. The incidence of CT or NG was defined as a new infection (i.e., conversion from a negative result to a positive result). A positive CT or NG result after treatment for the respective infection was regarded as treatment failure and not counted toward the incidence of CT or NG. The incidences of rectal and urinary tract CT and NG were estimated during the observation period between January 1, 2017 and December 31, 2018. The observation period for pharyngeal CT and NG was January 1 to December 31, 2018 because pharyngeal CT and NG testing was added after January 1, 2018.

This study was approved by the ethical committee at the NCGM (NCGM-G-002091-00), and all participants provided written informed consent in accordance with the Declaration of Helsinki. Regarding participants under the age of 18, the ethical committee approved the lack of parental or guardian consent.

### Statistical analysis

For categorical variables, Chi-square or Fisher’s exact tests were applied as appropriate. The independent t-test was used to compare means. Statistical significance was defined as a two-sided *p* value of < 0.05. All statistical analyses were performed using SPSS ver. 25.0 (IBM SPSS, Chicago, IL, USA).

## Results

### Prevalences of rectal CT and NG

We enrolled 502 MSM in the HIV-negative cohort between January 2017 and December 2018. Among them, 13 were diagnosed with HIV infection at enrollment and were excluded from subsequent analyses. We also cross-sectionally evaluated 574 HIV-positive MSM for the prevalences of rectal CT and NG between January and March 2018. Table 1 shows the prevalences of rectal CT and NG among MSM in Japan. HIV-negative MSM were significantly younger than HIV-positive MSM (p<0.001). The prevalences of rectal CT and NG were 16.1% (171/1,063) and 3.2% (34/1,063) for the HIV-negative and–positive MSM cohorts, respectively, which were not significantly different. Table 2 illustrates the prevalences of rectal, pharyngeal, and urine CT and NG among MSM in the HIV-negative MSM cohort for the first visit between January and March 2018, during which urine sample collection for CT and NG was cross-sectionally performed. During this period, 118 MSM were evaluated for rectal, pharyngeal, and urinary CT and NG and the prevalences were 16.9%/3.4%, 0.8%/2.5%, and 3.4%/0%, respectively. Rectal CT and NG infections were disproportionally higher than the respective pharyngeal and urinary infections (p<0.001). Of 27 MSM with CT or NG infection, multisite infections were observed in two cases. One case possessed rectal and pharyngeal CT infections and the other case had rectal and pharyngeal NG infections.

**Table 1:** Prevalences of rectal *Chlamydia trachomatis* and *Neisseria gonorrhoeae* among men who have sex with men in Japan.

	Total	HIV-positive MSM	HIV-negative MSM	P value
Number	1063	574	489	
Average age, years	40.4	46.2	33.6	<0.001
Rectal CT and/or NG n (%)	189 (17.8%)	99 (17.2%)	90 (18.4%)	0.623
Rectal CT	171 (16.1%)	91 (15.9%)	80 (16.4%)	0.235
Rectal NG	34 (3.2%)	14 (2.4%)	20 (4.1%)	0.161
Rectal dual infection	16 (1.5%)	6 (1.0%)	10 (2.0%)	0.086

MSM, men who have sex with men; CT, *Chlamydia trachomatis*; NG, *Neisseria gonorrhoeae*.

*P* values comparing continuous and categorical variables were determined using the Mann–Whitney *U* test and Fisher’s exact test, respectively.

**Table 2:** Prevalences of *Chlamydia trachomatis* and *Neisseria gonorrhoeae* according to infection site among HIV-negative men who have sex with men who were enrolled in the study cohort between January and March 2018.

Number	118
Average age, years	34.7
Rectal CT and/or NG n (%)	21 (17.8%)
Rectal CT	20 (16.9%)
Rectal NG	4 (3.4%)
Rectal dual infection	3 (2.5%)
Pharyngeal CT and/or NG n (%)	4 (3.4%)
Pharyngeal CT	1 (0.8%)
Pharyngeal NG	3 (2.5%)
Pharyngeal dual infection	0 (0%)
Urinary CT and/or NG n (%)	4 (3.4%)
Urinary CT	4 (3.4%)
Urinary NG	0 (0%)
Urinary dual infection	0 (0%)
Total CT and/or NG n (%)	27 (22.9%)

Data are expressed as number (%) of patients or mean (range).

CT, *Chlamydia trachomatis*; NG, *Neisseria gonorrhoeae*.

### Incidences of rectal, pharyngeal, and urinary CT and NG

Next, we evaluated the incidences of rectal, pharyngeal, and urinary tract CT and NG in the HIV-negative cohort. Of the 489 subjects in this cohort, 328 were followed at least twice, with a mean observation period of 290.5 days (261.1 person-years) between January 2017 and December 2018. To determine the incidences of pharyngeal CT and NG infections, we observed subjects who were enrolled in the HIV-negative cohort on and after January 2018. Table 3 shows the incidences of rectal, pharyngeal, and urinary CT infection were 17.2 (45 cases)/100 person-years, 2.0 (2 cases)/100 person-years, and 0.4 (1 case)/100 person-years, respectively. The incidences of rectal, pharyngeal, and urinary NG infection were 3.8 (10 cases)/100 person-years, 11.0 (11 cases)/100 person-years, and 1.9 (5 cases)/100 person-year, respectively. Regarding incidental CT or NG among 10 MSM who became infected with HIV, three cases of rectal CT or NG cases with 5.62 person-years (53.4%/year) were observed, which was higher than the total number of similar cases in the HIV-negative cohort. Recurrent infection was common among HIV-negative MSM in this cohort. Of 66 MSM who became infected with CT or NG during the observation period, 25 (37.9%) MSM had recurrent infections, 22 (88.0%) of whom had experienced rectal CT and/or NG infection. As for co-infection of CT and NG among the incidental cases, only two cases (0.77%/year) of rectal co-infection were observed in the cohort.

**Table 3:** Incidences of rectal, pharyngeal, and urinary *Chlamydia trachomatis* and *Neisseria gonorrhoeae* among HIV-negative men who have sex with men in Japan.

	New incidental case (n)	Incidence rate/ 100 person-year
HIV*	10	3.8
Rectal CT and/or NG*	55	21.1
Rectal CT	45	17.2
Rectal NG	10	3.8
Pharyngeal CT and/or NG**	13	13.0
Pharyngeal CT	2	2.0
Pharyngeal NG	11	11.0
Urinary CT and/or NG*	6	2.3
Urinary CT	1	0.38
Urinary NG	5	1.91

CT, *Chlamydia trachomatis*; NG, *Neisseria gonorrhoeae*.

*Mean observation period = 290.5 days (328 MSM, 261.1 person-years)

**Mean observation period = 202.3 days (182 MSM, 99.8 person-years)

## Discussion

In the present study, we determined the prevalences of rectal CT (16.1%) and NG (3.2%) among MSM in Japan. We also illuminated the incidences of rectal, pharyngeal, and urinary CT and NG in a prospective cohort among HIV-negative MSM. The prevalence of rectal CT (16.1%) was considerably higher than the mean prevalence (9%) reported in a recent meta-analysis[7], which suggests that rectal STIs have been neglected in Japan. In addition, a study of over 10,000 MSM who presented to an STI clinic reported that 14% of these MSM were positive for rectal CT[5]. Notably, most MSM in our study were asymptomatic and not aware of their STI status. Our results strongly suggested that only urine CT and NG testing leads to missing most infected cases, as previously reported[5,11–13]. Thus, rectal and pharyngeal CT and NG infections require urgent attention in Japan. From this standpoint, a recent study showed the utility of triple-site pooled sample collection into a single sample (referred to as a “3 in 1 study”) as a cost-saving measure[14], which could be useful to decrease the number of missed diagnoses.

Regarding rectal NG, our study showed a relatively low prevalence compared with the previously reported median prevalence (5.9%)[15]. Similar to this, the prevalence of pharyngeal NG in our study was just 2.5%, which was lower than the previous review that showed a median prevalence of 4.6% among MSM[15]. There was a large discrepancy between the high prevalence of rectal CT and the low to moderate prevalence of pharyngeal NG in our study. One potential explanation for why the prevalences of rectal and pharyngeal NG were relatively low in our study could be attributed to the fact that symptomatic urethritis caused by NG was more likely to be treated before NG was transmitted than urethritis caused by CT, which might be asymptomatic. Interestingly, our study showed that the prevalence of rectal CT in HIV-negative MSM was higher than that in HIV-positive MSM, which was inconsistent with previous studies that showed higher prevalences of rectal STIs in HIV-positive MSM than in HIV-negative MSM[16]. This could be affected by the significantly younger mean age of the HIV-negative MSM cohort in this study.

The incidences of rectal CT and pharyngeal NG were found to be 17.2/100 person-years and 11.0/100 person-years, respectively. The incidence of total CT and NG (36.4/100 person-years) in our cohort was higher than the 20.8/100 person-years reported as the incidence of all asymptomatic multisite STIs in an earlier study[17]. Notably, recurrent CT and NG infections were common and as high as 37.9% among MSM infected with CT or NG. Of these MSM with recurrent infections, 88% experienced rectal CT or NG infection, which implies that routine CT and NG testing, including rectal sampling, is justified among asymptomatic MSM. Previous studies suggested that rectal infection should be evaluated even when there is no reported history of receptive anal sexual intercourse not only because some MSM might conceal their sexual activity but also because some individuals may use saliva as an anal lubricant, which could be associated with STI transmission from pharyngeal to rectal sites[18,19].

This study has some limitations that should be considered. First, this was a single-center prospective study with a relatively short observation period. The sample size was relatively large for evaluation of the prevalences of rectal CT and NG. However, the incidences of CT and NG should be examined over a longer time period, especially regarding pharyngeal CT and NG. Second, the presence of CT or NG detected after treatment was regarded as treatment failure and was not counted toward the incidence of CT or NG, which could lead to underestimation of the incidences of CT and NG. Third, urethral CT and NG were also likely underestimated in this study since urine testing for CT and NG was conducted at the physician’s discretion when MSM were asymptomatic or reported sexual intercourse with partners with STIs. Indeed, the incidence of urethral CT or NG among MSM was 2.3/100 person-years, which is lower than previously reported[20]. In general, our study had a tendency to underestimate the incidences of CT and NG, which could fail to reflect the real situation regarding urethral CT and NG among MSM in Japan. Fourth, the serotypes of CT were not initially evaluated in this study population, however, we commenced serotype analysis in September 2018 in the non HIV-infected MSM cohort. Of 67 MSM who were diagnosed with CT infection, 42 were successfully analyzed for their serotypes. Most of the serotypes were G, J/Ja and D/Da, whereas serotype L, *Lymphogranuloma venereum* (LGV) was not observed in this cohort (unpublished preliminary data from Dr. Miyake, Tokyo Metropolitan Institute of Public Health).

In conclusion, our results suggested that the prevalence and incidence of rectal CT infection were considerably higher than those of pharyngeal and urinary CT infection among MSM in Japan, which could be partly attributed to a lack of awareness of the presence of rectal STIs among MSM. Urgent countermeasures are required to establish an effective rectal CT and NG testing system in Japan for the further implementation of preventive strategies against HIV and otherSTIs, including PrEP.

## Supporting information

S1 Dataset Data sources for prevalence of Chlamydia and gonorrhoeae among HIV-infected men who have sex with men in the Table 1. (XLSX)

S2 Dataset Data sources for prevalence of Chlamydia and gonorrhoeae among non HIV-infected men who have sex with men in the Table 1. (XLSX)

S3 Dataset Data sources for the Table 2. (XLSX)

S4 Dataset Data sources for incidence of rectal and urinary Chlamydia and gonorrhoeae in the Table 3. (XLSX)

S5 Dataset Data sources for incidence of pharyngeal Chlamydia and gonorrhoeae in the Table 3. (XLSX)
